# Tobacco

**Published:** 1893-04

**Authors:** S. H. Preston


### TOBACCO.

By S. H. Preston.

Everybody has heard of the celebrated counterblast of James I. against tobacco—how he denounced it as an incentive to drunkenness, heaped upon it the heaviest taxes, and finally forbade the use of it in ale houses. Nothing aroused the anger of the Scotch King so much as a whiff of tobacco smoke. Tobacco was not tolerated in his dominions except as an expensive medicine.

But few are aware of the fact that it once was the provocation of Papal Bulls, and that its use was made a capital crime by the edicts of Sultans and Czars. In 1684, Pope Urban VIII. published a bull excommunicating all persons found guilty of taking snuff in church. This bull was renewed by Pope Innocent, in 1690. Twenty-nine years later the Sultan Amarath IV. made smoking and snuffing punishable by death. The punishment of snuff-takers in Russia was the loss of their noses. The same punishment was enforced against smokers, and for the third offence they lost their lives. In Switzerland the laws were nearly as severe, and in 1661 an addition was made to the ten commandments to be read in all the churches throughout the cantons. The new commandment followed the seventh, and forbade the taking of tobacco in any form as a deadly sin.

From the first the church seems to have scented out the agency of Satan in smoking and snuffing tobacco. It was found that it destroyed the efficacy of fasting. The Puritans set their faces against it the most sternly of any of the sects. Their bigoted bitterness against it was partly based on the ground that it was nowhere mentioned in the Bible, but principally because of the comfort it afforded the profane people who indulged in its use.

But despite all the rigors of restrictive duties, the denunciations of priests, the bulls of popes and the edicts of emperors; despite fines and tortures and death penalties, the use of tobacco everywhere continued to increase. For thirty years the tobacco traffic thrived to an extraordinary extent all over Europe. Aubrey says that the poisonous plant sold for its weight in silver in Great Britain, and that he had heard old yeomen boast that they had culled their biggest shillings to bear down the scales in buying tobacco.

In those times it was usually used in the form of snuff. A story is told of a rich old snuff-taker named Mrs. Thompson, who died in London in 1776, leaving with each legacy specified in her will "one pound of good Scotch snuff." She had declared up to the day of her death her faith in it as the "grand cordial of Nature." In her day Scotch snuff had acquired great popularity, and nearly everybody used it, especially the ladies. Snuffing was then the rule, smoking the exception. Napoleon, Pope, Voltaire, Steele and Lady Wortley Montague were all notorious snuff-takers.

But a fact not as well known to many readers as the foregoing is that the first exportation of tobacco from this country was for the purchase of white women for wives. The colony at Jamestown had been established thirteen years when a want was felt for more women. Ninety respectable females were imported from England and sold to the Jamestown planters at the rate of 120 pounds of tobacco each. In 1621, seventy more wives were sent over and bartered for tobacco, but this time at the rate of of 150 pounds per head. The London Company had got a "corner" in the women and tobacco trade.

And thus tobacco becomes associated in our history with slave-holding and wife buying. Estimating crops for what they would buy, a field of tobacco in those times might have been called worth so many wives. Had the statistician been around in those days we might have been told how many wives worth of tobacco were smoked and chewed up annually.

So that women especially have a justifiable grudge against this execrable evil, as it first makes a slave of man, and then man makes woman his slave. It makes man a beast, and then makes woman a victim of that beast.

The evil effects of tobacco on health are too well known to refer to here at length. The truth is not now questioned that it not only originates certain complaints, but that it greatly aggravates those that come from other causes. About the only apology for its use is that it stimulates the intellect. The prominence of the pipe in literature is triumphantly pointed to in proof of this statement. It is observed that Lord Bacon has eulogized it; that Drummond, Fletcher, Beaumont, Ben Johnson, Sir Isaac Newton, Addison, Dryden, Defoe, all the brilliant men of Queen Anne's day, Sir Walter Scott, Dickens, Tennyson, Spurgeon, Longfellow, Lowell, Aldrich, Clemmens, Bismarck, all advocate the pipe.

An equally illustrious list of literati, including James Parton, who wrote much on the subject, earnestly entreat brain workers to discard tobacco. No medical man ever maintained that the mental faculties could be quickened to any utility by tobacco. The intellectual operations may be momentarily stimulated by tobacco smoke, the same as by strong drink; but it is a noticeable fact that old tobacco users think more tardily than others. There are enough authorities showing the irreparable injury of tobacco to the brain as well as the body to fill a volume; but the opinion of Prof. Hitchcock must suffice for present space. He says :

"Intoxicating drinks, opium and tobacco exert a pernicious influence upon the intellect. They tend directly to debilitate the organs ; and we cannot take a more effectual course to cloud the understanding, weaken the memory, unfix the attention, and confuse all the mental operations, than by thus entailing upon ourselves the whole hateful train of nervous maladies. These can bow down to the earth an intellect of giant strength, and make it grind in bondage, like Samson shorn of his locks and deprived of his vision. The use of tobacco may seem to soothe the feelings and quicken the operations of the mind; but to what purpose is it that the machine is furiously running and buzzing after the balance wheel is taken off."

